# Posture correctness of young female soccer players

**DOI:** 10.1038/s41598-019-47619-1

**Published:** 2019-08-01

**Authors:** Beata Żuk, Marek Sutkowski, Sławomir Paśko, Tomasz Grudniewski

**Affiliations:** 1 Department of Biophysics and Human Physiology, Medical University of Warsaw, Chałubińnskiego 5, 02-004, Warsaw, Poland; 2 Institute of Microelectronics and Optoelectronics, Warsaw University of Technology, Nowowiejska 15/19, 00-665, Warsaw, Poland; 3 Institute of Micromechanics and Photonics, Warsaw University of Technology, A. Boboli 8, 02-525, Warsaw, Poland; 4 Faculty of Technical Sciences, State School of Higher Education, Sidorska 95/97 21-500 Biała, Podlaska, Poland

**Keywords:** Musculoskeletal system, Imaging and sensing, Bone quality and biomechanics

## Abstract

The objectives of the study were to evaluate the correctness of the body posture of female soccer players in the frontal plane from the back based on selected body points in two static positions (habitual and actively corrected) using a non-contact optical measurement method. Forty-two young women (aged 16–20) playing soccer in a sports club in Poland were examined and compared with controls. The spatial coordinates (x, y, z) of the selected body points were determined. Four points (Oc_L_, Oc_R_, Pv_L_ and Pv_R_) were extracted and used to calculate vectors $\overline{{\boldsymbol{Oc}}}={\boldsymbol{O}}{{\boldsymbol{c}}}_{{\boldsymbol{L}}}-{\boldsymbol{O}}{{\boldsymbol{c}}}_{{\boldsymbol{R}}}$ and $\overline{{\boldsymbol{Pv}}}={\boldsymbol{P}}{{\boldsymbol{v}}}_{{\boldsymbol{L}}}-{\boldsymbol{P}}{{\boldsymbol{v}}}_{{\boldsymbol{R}}}$ for analysis. The results show that median of the pelvic line angle was positive (Pv_R_ was lower than Pv_L_) in both groups. For the habitual posture, the absolute value of the difference between the 25th and 75th percentiles in the pelvic line was almost three times greater among the soccer players than the controls (ratio between soccer players and controls: 2.93). Static postural imbalances in female soccer players require diagnosis of the sacroiliac joints with analysis of lumbar-pelvic system support and inhibition in the context of myofascial connection integration. Exercises can be implemented to stabilize the lumbar-pelvis complex as prophylaxis for spinal overload during the training cycle.

## Introduction

The intuitive habitual standing posture a human maintains against the force of gravity is the external manifestation of the spatial arrangement of particular body segments. This posture is subject to continuous regulation via reflexes due to feedback, mainly originating in proprioceptors found in the networks of muscles, tendons, and joint capsules. These proprioceptors constantly provide information to the central and peripheral nervous systems about the current posture, the tension and length of muscles, and the position of the trunk and limbs in relation to each other. Body posture is not a passive system consisting of individual segments; rather, the act of movement is distinguished by a high level of automation, followed by a coded postural pattern. It is the static position of the body that begins and ends all motor activities^1–3^.

The body posture of sportsmen is an area of interest for many researchers seeking to determine potential relations between body asymmetry and unilateral load accumulation, as well as specific injuries^4–7^. Volleyball is an example of a discipline associated with high training loads. The sport involves a number of asymmetrical techniques, including the serve and the attack. Volleyball can result in an imbalance between the muscle tonus and length and thus contribute to asymmetry of the spine. Relevant observations have been described by Kugler *et al*. (1996), Vařeková *et al*. (2011), Loffing (2012), and Grabara (2015), among others^8–11^.

Women’s soccer is a relatively young sport discipline. Similar to men’s soccer, it is an endurance sport with repetitive complex motion sequences, as well as a high risk of body injury^12^. The frequency of injuries among adult male players is estimated at 10–35 per 1000 hours of activity. Approximately 60–80% of all serious injuries occur in the lower limbs. In most cases, these injuries involve damage to the anterior cruciate ligament (ACL). Jakobsen and Tenger, in their analysis of a group of 224 female soccer players (age 23 ± 4, height 168 ± 5 cm, weight 62 ± 7 kg, body mass index [BMI] 22 ± 2), observed more injuries to the knee than to other areas of the body^13^. Brophy *et al*. showed that women playing soccer tend to have non-contact knee injuries in the preferred supporting leg, while men tend to have injuries in the limb leading the ball^14^. As the authors suggest, reasons for these divergences could be related to anatomical differences and/or sex-specific neuromuscular control. Moreover mechanics of the lumbar-pelvic-hip complex with the dorsal surface of the thoracolumbar fascia participate in the transfer of loads between the lower and upper limbs, the spine and the pelvis. Therefore, the analysis of possible pelvic asymmetries in posture measurements will also reflect the changes in human motor system caused by injuries in the lower limbs. The presented results can be considered a foundation for this work, but there is no doubt that this problem requires further research.

In the literature related to sports medicine, there are only few reports on the assessment of player posture and on female players in general. Various training methods for soccer (e.g., strength, speed, and interval training) affect the body’s performance but do not influence the body’s posture in all planes equally. Grabara analysed the body posture of young soccer players aged 11–14 and their untrained peers in the sagittal, frontal and transverse planes using computer posturography (Moiré Shadow Technique). Obtained results show a greater symmetry of the pelvis in the frontal plane of the back for trainers with a greater tendency towards asymmetry of the shoulder blades than their non-training peers. However, the author drew attention to the ambiguity of the obtained results^15^.

## The Aim of Study

The aim of the present work was to evaluate the correctness of female soccer players’ body posture in the frontal plane from the back based on the selected body points in two static positions, i.e., habitual and actively corrected positions, using a non-contact optical measurement method.

## Methods

This paper includes the results of pilot studies carried out in March 2017 at the State School of Higher Education in Biała Podlaska, Poland. A total of 57 women (aged 16–20) were examined, including 42 sport club players (AZS PSW Biała Podlaska, classified in Ekstraliga Kobiet – Polish female Premier League) and 15 non-players as controls (by random selection). This study was approved by the bioethics commission of Warsaw Medical University (number: KB/49/2017), and all research was performed in accordance with relevant guidelines. Before the body posture measurement process began, each participant was informed about the purpose, voluntariness, and non-invasiveness of the study and asked to provide written consent to participate. Consents are signed personally and by parents or legal guardians in the case of participants under the age 18 y.o. Additionally, each participant completed a specially prepared questionnaire consisting of 16 closed questions for soccer players and 9 closed questions for the controls. The questions collected information regarding age, height, body mass, education level, residential area, pain experienced over at least a week before the study, and the most preferred type of physical activity during the year prior to the study. The soccer players were also asked about the time they spend at regular soccer training sessions at the sports club, the frequency of practice, and the preferred arm and leg used during soccer play and to specify the location and type of any injuries suffered during the past year. The participants in the control group were randomly selected, and none of them declared any primary physical activity. Only 2 participants declared participation in systematic physical training, one trained at a gym, and 7 declared bike rides. Thus we can assume controls as a group of women not practicing sport regularly.

The general characteristics of the test groups are shown in Table 1.

**Table 1:** Selected physical, sport and demographic data by group (mean ± SD).

		Socc (n = 42)			Ctrl (n = 15)	p-value
Weight [kg]		58.3 ± 6.7			55.5 ± 7.5	0.012
Height [cm]		166.8 ± 5.8			165.7 ± 4.8	0.238
BMI		21.0 ± 2.3			20.2 ± 2.6	0.006
Duration of playing sport	[yr.]	≥6	3–5	<3	n.a.	n.a.
	[%]	50	45.2	4.8		
Number of trainings per week	[a.u.]	5–7	3–4	<3		
	[%]	57	33	10		
Duration of trainings	[h]	>3	2–3	<2		
	[%[	0	93	7		

Socc – soccer players, Ctrl – controls, p < 0.05 (two-sided non-parametric Mann-Whitney-Wilcoxon).

The weight, height and BMI data were not normally distributed in either group, as determined by the Shapiro-Wilk test; therefore, the two-sided non-parametric Mann-Whitney-Wilcoxon (known also as Wilcoxon rank sum) test was used for the assessment. The significance level (p-value) was set at 0.05 by default. Apart from height, the other parameters differed significantly between the groups.

The group of soccer players was dominated by girls with a primary education (78%) living in cities (10,000–100,000 inhabitants); in the control group, over half of the participants had a secondary education (60%) and lived in the countryside (53%). Table 1 shows sport data regarding the soccer player group (duration of practicing sport, number of trainings per week, duration of trainings).

Among the soccer players, 31 women declared the right lower limb to be dominant, 7 reported the left leg to be dominant, and 4 reported equal use of both legs in leading the ball. Regarding the upper limbs, 37 players were right-handed, and 5 were left-handed. A third part of the players (33.3%) declared injuries. Nine women had foot injuries, and 5 had knee injuries, but none of the players reported movement-related discomfort the week before the study.

The photogrammetric evaluation of posture was performed with the participant in underwear and a silicone head cap. Each subject stood in a relaxed position with their feet slightly spaced inside the calibrated measuring space with their back turned towards the cameras. The lower limbs were straight, the arms were loose along the torso, and the gaze was fixed on one point straight ahead. Special circular markers with a white central field (φ = 10 mm) and black edges were applied to specific points on the body for the examination; 17 bone points on the torso and lower limbs were marked. The points were selected on the base of known methods for collecting body topography measurements^1,4–7^. As this paper presents an analysis of body asymmetries in the frontal plane of the back, only specific points were extracted.

**Figure 1:**
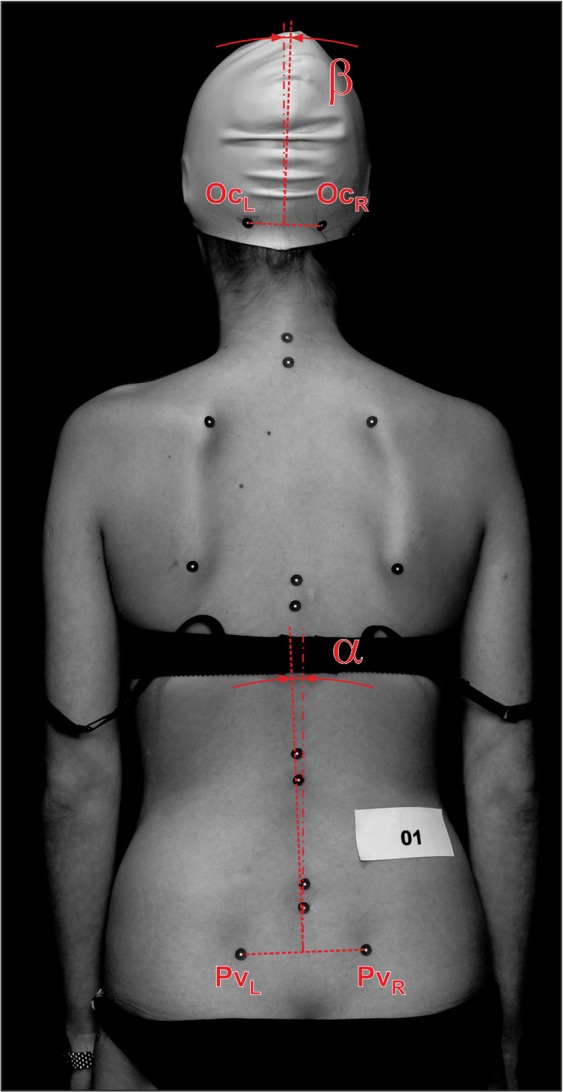
Method for determining parameters for analysis. Oc_L_, Oc_R_ - superior nuchal points of the skull (left and right); Pv_L_, Pv_R_ - rear upper iliac spines (left and right); α - angle between vector $\overline{Oc}$ and vertical; β - angle between vector $\overline{Pv}$ and vertical. NOTE: The image above is for information only and was not used for measurements or analysis.

The Oc_L_ and Oc_R_, i.e., the left and right superior nuchal points of the skull (left and right) and the Pv_L_ and Pv_R_, i.e., the left and right rear upper iliac spines (see Fig. 1) were used to calculate the vectors $\overline{Oc}=O{c}_{L}-O{c}_{R}$ (1) and ${\bar{P}}_{v}=P{v}_{L}-P{v}_{R}$ (2) and determine the pelvic angle (inclination angle of the pelvis) and the inclination angle of the extreme points of the upper and perpendicular margins. A negative value for the determined angle indicated a lowering of the left bone point (Oc_L_ or Pv_L_) relative to the corresponding right bone point (Oc_R_ or Pv_R_). The same statistical method as for weight and BMI was used to compare both groups results. To assess the relationship between the aforementioned angles and categorical data, e.g. position on the pitch, one-way ANOVA was used.

Measurements were collected for two positions: the resting stance (habitual) and an actively corrected position (i.e., at the verbal command, “attention”). The subjects were not informed about slight irregularities between habitual standing postures and those possible with global body corrections. For them, their intuitive activation of the upright position was derived from reflexes based on their concept of spinal stabilization.

## Results

Assessment of the correctness of the examined postures demonstrated differences in the pelvic position’s symmetry and a tilt between the upper points (Table 2). In the resting position, a greater pelvic inclination angle was observed in the soccer players than in the controls (larger values of mean and median in both postures). The absolute difference between the 25th and 75th percentiles was nearly three times greater among the soccer players than among the controls, with a ratio of 2.93. Different values of standard deviations were shown in the slope of the upper points, i.e. greater standard deviation were observed in the controls than in the soccer players; here, the ratio calculated on absolute difference between the 25th and 75th percentiles (|Q25–Q75|) for the groups, which were 2.95 and 2.45 respectively was 1.20. In both groups, the median pelvic angle was positive, indicating a lower position for Pv_R_ in relation to Pv_L_.

**Table 2:** Inclination angles in habitual and actively corrected postures.

[°]	Inclination angle of the pelvis				Inclination angle of superior nuchal line			
	Habitual		Active		Habitual		Active	
	Socc	Ctrl	Socc	Ctrl	Socc	Ctrl	Socc	Ctrl
min:	−2.83	−0.81	−2.62	−0.76	−5.83	−6.77	−5.19	−7.97
max:	7.26	4.01	7.37	3.87	3.06	4.59	2.24	3.05
range:	10.09	4.82	9.99	4.63	8.89	11.36	7.43	11.02
mean (SD):	3.51 (±2.13)	2.14 (±1.29)	3.51 (±2.12)	2.19 (±1.33)	−1.29 (±2.07)	−2.32 (±2.64)	−1.37 (±1.94)	−1.81 (±2.77)
median:	3.47	2.02	3.61	2.24	−1.18	−2.28	−1.34	−1.94
Q25	2.13	1.89	2.28	1.91	−2.47	−4.10	−2.44	−3.33
Q75	5.12	2.91	4.63	3.17	−0.02	−1.15	−0.41	−0.17
|Q25-Q75|	2.99	1.02	2.35	1.26	2.45	2.95	2.03	3.16
p-value	0.011		0.016		0.016		0.016	

Description: Socc – soccer players, Ctrl – controls, p < 0.05 (two-sided non-parametric Mann-Whitney-Wilcoxon), range = max – min; Q25 – 25% percentile, Q75 – 75% percentile, SD – standard deviation.

Regarding the tilt of the superior nuchal line tilt (median), the results were similar, except that in both groups, Oc_L_ was in a lower position relative to Oc_R_. The inclination angle of superior nuchal line is characterised by a noticeably smaller tilt for players as for controls (median and mean), just opposite as for the pelvis line. Moreover in the group of soccer players we observe a slightly increased tilt for active corrected posture and decreased for controls.

The same parameters were analysed with each subject in an actively corrected upright position, which was assumed immediately after the first set of measurements. The obtained results are summarized in Table 2, highlighting the small angular changes in both groups according to the habitual position (median is approx. 0.14–0.22° larger for active corrected posture).

To explain the differences between the players’ posture and that of the controls, further analysis was performed regarding the position on the pitch and the preferred lower limb.

In both examined the habitual and actively corrected postures, the same correlation coefficient (r = 0.03 – taken as one-way ANOVA) was found between the pelvic angle and the position on the pitch. However, the pelvic position was not correlated with the preferred limb used during the game for either the habitual (r = 0.76) or actively corrected posture (r = 0.69).

Interestingly, the angular asymmetry of the head position and the lower limb preferred by the player showed a correlation coefficient of r = 0.08 for the habitual posture and a coefficient of r = 0.04 for the actively corrected posture.

## Discussion

The presented body posture analysis of a small representative group of female soccer players seems to be an extension of the investigations led by many researchers interested in human posture asymmetry resulting from unilateral training loads. However in this section we compare results of examination to previous investigations, but direct analysis is very controversial due to the lack of sufficient reports regarding strictly female soccer players, a sport discipline with relatively small popularity (resulting in small population practicing it worldwide). We noticed few consistencies and inconsistencies of our findings with research results reported by other authors for sportsmen of other sex and disciplines.

Previous research using a non-contact 3D photogrammetry system provided the basis for further analysis of changes taking place in the posture of the body under the influence of physical activity (in soccer players, these changes are dependent on the different loads of movement mechanisms)^16,17^.

### The pelvic

The pelvic rim is involved in the transmission of forces between the spine and the lower limbs, and in terms of both static and dynamic forces, it constitutes a significant closed kinematic chain with the upper cervical section of a human spine.

In the habitual posture, the level of pelvic asymmetry (lower Pv_R_ vs Pv_L_) among the soccer players was almost triple that among the controls, demonstrating rotational changes in the pelvic rim on the horizontal plane, oriented to the vertical axis (y). The cause of these changes, which changed little with active correction, could be disordered movement patterns originating from the hip^18^. It is assumed that Pv_R_ lowering is accompanied by the backwards inclination of the right iliac plate, with modifications to the dynamic connections among ligaments and muscles. Due to the static equilibrium of the pelvis, the position of Pv_L_ changes accordingly, with an inclination of the iliac plate towards the front.

The observations above are in agreement with those of Hidges *et al*., who used magnetic resonance imaging (MRI) of the psoas major (PM) and quadratum lumborum (QL) muscles to show their asymmetry towards the preferred limb. In 54 Australian Soccer League (AFL) athletes, these researchers discovered that the cross-sectional area (CSA) of the psoas of the limb leading the ball was much greater than that on the opposite side, in which the quadratus lumborum muscle showed the greatest CSA. The asymmetry of the muscle size was not correlated with the number of injuries^19^. Other studies examining the asymmetry of the pelvic muscles in relation to the dominant limb, have drawn attention to the impairment of deep lumbar spine stabilizers (in soccer players without LBP – low back pain), mainly the multifidus (MF) and transversus (TrA) muscles, and suggested the need to implement lumbar-pelvis complex stabilization exercises^20,21^.

A correlation was found between the pelvic angle and the position of the players on the pitch but not between the pelvic angle and past injuries incurred during training or during a game. The pelvic angle might have prognostic value for determining a female athlete’s susceptibility tendon and muscle injuries.

Elliott *et al*. conducted 10 years of research based on information gathered from soccer coaches (registered with Niel’s Injury Surveillance System) and showed a higher rate of injuries among defensive male players (28.1%), mainly during the 7 preseason weeks^22^ but no reports were found in the literature regarding the relations among changes in the spatial position of the pelvis, the tendency to sustain injuries in the lower limbs and the position of female players. Thus it is risky to predict any relationship between pelvis parameters and female player’s injury threat.

### Lower nuchal limits

Other important observations for evaluating the global posture of the body, which can be considered a complex tensegritous construct, are changes in the inclination angles of the extreme lower nuchal limits, coexisting with changes in the pelvic inclination in both positions and both groups. It is worth noting that changes in the determined angles in the cervical posterior region were almost three times greater among the soccer players compared with controls which may indicate postural errors; however, the diagnosis of such errors was not the purpose of the study.

The results show that among the female soccer players, there was a correlation between the angle determined by the extreme lower nuchal limits (Oc_L_ vs Oc_R_) and the dominant lower limb (right limb in 88.1%), which coincides with the theory of Ingber, indicating that increased tension in one of the limb increases the tension in other elements of the body, even those on the opposite side^23^.

### Functional tapes

The concept of the body consisting of a series of structures with systemic connections was developed by others, including Stecco A, Myers T, and Stecco C, who examined the function of the fascia in terms of micro- and macrotensegrity^24–26^. Fascia, a type of connective tissue that penetrates and surrounds all organs, muscles, bones, and nerve fibres, has the ability to transfer biomechanical forces by changing its shape in response to loads or adaptation to long-term loads at the level of microtensegrity. In soccer, the loads are associated with taking the ball from the opponent and stretching the side structures of the body with a reciprocal twist of one of the anatomical tapes surrounding the body in a double helix shape (according to Myers’ spiral and functional tapes).

Due to the lack of reports, it can be argued that the body posture of the tested soccer players is characterized by myofascial disorders of the spiral tape’s initial part (lower Oc_L_ vs Oc_R_) and of the deep frontal tape cooperating with it, with the PM and QL muscles affecting the position of the pelvis (lower Pv_R_ vs Pv_L_) and cervical segments of the spine muscles (balancing the strokes of the superficial tapes anterior and posterior of the cervical segment of the spine). The spiral course of the myofascial connections is also characterized by a functional tape (back and front), most often activated during sports activities. Although it does not play a significant postural function, it can create a compensation pattern associated with a preferred and repetitive direction of movement.

### Dominant limb

The vast majority of the examined players indicated the right lower limb to be dominant, i.e., more loaded during matches and training. Therefore, one can posit that to increase the moment of force while kicking the ball with the right leg and maintain the body’s balance, players more often tend to lift the left upper limb upward and forward (at the same time extending the widest spine muscle on the left)^25^. The correlation between the preferred lower limb (right) and the Oc_L_ angle, demonstrated in this research, is the result of a unilateral change in the latissimus dorsi muscle potential, the path of tension transmission between a shoulder and the pelvic rim on the right and left sides of the body. This correlation may also represent a response to lower the dynamic loads of body areas with greater CSAs and/or the result of an imbalance between the length and strength of hip flexors and extensors. McLean (1993) noted the essence of lower limb muscle imbalance problems in soccer^27^. Bussey (2010), Ekstrand (2012) and Stecco (2014) did not refer to global changes in body symmetry^28–30^. There is a lack of reports in the literature on the relation between the position of the posterior cervical region and the dominant limb, which makes it impossible to unequivocally assess the reported changes.

## Conclusions

This analysis of the body posture of soccer players vs controls using a 3D photogrammetry measurement system accurately illustrates the differences between habitual and actively corrected upright postures. The continuation of this research is critical, considering the impact of the foot and knee on the postural pattern. The presented results can be summarized as practical conclusion: exercises can be implemented to stabilize the lumbar-pelvis complex as prophylaxis for spinal overload during the training cycle.

### Practical Implications

- Based on the present results, it is recommended to analyse each player’s movement system with particular emphasis on the position of the pelvis and skull in both habitual and actively corrected postures.
- Body asymmetries (e.g., as noted in the paper) may be caused by one-sided training loads, the pitch position or the preferred lower limb. The implementation of exercises to reduce myofascial imbalances in the musculoskeletal system and core stability is justified.
- Analysis of body posture parameters is necessary to evaluate postural changes in players and to prevent injuries. Optical measurement systems (including 3D photogrammetric systems) can be very helpful, especially due to their ability to reveal errors in body posture, as well as individual compensation patterns.
- Static imbalances in the body posture of female soccer players requires the diagnosis of the sacroiliac joints with analysis of lumbar-pelvic system support and inhibition in the context of myofascial connection integration.

The datasets generated during and/or analysed during the current study are available from the corresponding author on reasonable request.
